# Frontiers in Oral Mucosal Immunity and the Microbiome

**DOI:** 10.3389/froh.2021.821148

**Published:** 2022-01-03

**Authors:** Georgios N. Belibasakis, George Hajishengallis

**Affiliations:** ^1^Division of Oral Diseases, Department of Dental Medicine, Karolinska Institutet, Stockholm, Sweden; ^2^Department of Basic and Translational Sciences, School of Dental Medicine, University of Pennsylvania, Philadelphia, PA, United States

**Keywords:** oral mucosal immunity, oral microbiome, oral disease, oral infection, oral and systemic health

## Abstract

The 2nd International Conference on Oral Mucosal Immunity and the Microbiome (OMIM) took place at the Grecotel Kos Imperial Hotel, Kos, Greece, between 25th and 30th September 2021, under the auspices of the Aegean Conferences. This has only been the second Aegean Conference of this thematic, the first one having taken place in 2018 in Crete, during the same period of the year. Given the hardships in travel and heightened infection transmission risks amid the COVID-19 pandemic, the Conference was well attended by 29 international speakers across the world. For many of the participants, this was the first conference travel in the post-pandemic era, and quite significant that it has taken place on the island of Hippocrates. Stringent regional health and safety regulations had to be followed to accomplish for this in-person Conference to take place. Frontiers in Oral Health has hosted papers from presentations of the Conference, whereas the present article serves as the proceedings of the Conference with summaries of the presentations.

## Summaries of the Scientific Presentations

**Richard Lamont** addressed the multidimensional and context-dependent interactions between *Porphyromonas gingivalis* and *Streptococcus gordonii* which can occur through several mechanisms: (i) chemical communication, mediated by the streptococcal metabolite para-aminobenzoic acid (pABA), which can dampen the pathogenic potential of *P. gingivalis* by regulating gingipain production; (ii) in contrast, direct contact interactions between the two organisms can increase the pathogenic potential of the community *via* a tyrosine phosphorylation/dephosphorylation signaling cascade; (iii) *S. gordonii* can modulate the impact of *P. gingivalis* on epithelial cells using the host cell as an intermediary. Collectively these findings show that the output of the *S. gordonii*-*P. gingivalis* interaction is context dependent and may act as an accelerator or a brake on pathogenic potential.

**Marvin Whiteley** spoke about a framework for quantifying the accuracy of model systems used to study *P. gingivalis*. He showed that 96% of *P. gingivalis* genes were expressed similarly in human periodontitis and *in vitro* mid-logarithmic growth, while significantly fewer genes were expressed similarly in periodontitis and *in vitro* stationary phase cultures (72%) or in a murine abscess infection model (85%). This high conservation in gene expression between periodontitis and logarithmic laboratory growth is driven by overall low variance in *P. gingival*is gene expression, relative to other pathogens including *Pseudomonas aeruginosa* and *Staphylococcus aureus*. He proposed the use of simple test tube growth as the gold standard model for studying *P. gingivalis* biology, providing biological relevance for the thousands of laboratory experiments performed with logarithmic phase *P. gingivalis*.

**Julian Naglik** spoke about *Candida albicans*, one of the most widespread human fungal pathogens that can cause superficial mucosal but life-threatening systemic infections. Recently, the Naglik group showed that *C. albicans* produces a peptide toxin called candidalysin, which accounts for how this fungus damages host cells and induces immune responses. Upon secretion, candidalysin intercalates into and destabilizes the structural integrity of epithelial cell plasma membranes. As a result, alarmins are released and cell stress is induced, which leads to oral epithelial activation *via* two distinct MAPK pathways, namely, ERK1/2 and p38. Activation leads to the induction of a strong inflammatory response, mediated by neutrophils and natural Th17 cells, which protects against oral fungal infection.

**Keith Kirkwood** discussed about the role of periodontal disease in oral-gut transmission of pathogenic *Haemophilus parainfluenzae* in inflammatory bowel disease (IBD) patients. The pathogenicity of *H. parainfluenzae* was established using both acute and chronic mouse models of IBD. Correlation analysis using clinical data showed that periodontal disease in IBD patients potentiated the gut colonization by pathogenic *H. parainfluenzae* strains. In addition, clinically relevant pathogenic *H. parainfluenzae* strains synthesized colitogenic putrescine and disrupted the anti-inflammatory spermidine-hypusination pathway. Finally, analysis of the publicly available multi-omics data of IBD patients showed increased luminal putrescine level and bacterial ornithine decarboxylase transcription activity, which was strongly associated with the presence of *H*. *parainfluenzae*.

**Timo Sorsa** highlighted the need for reliable complementary adjunctive chair-side/point-of-care (POC) diagnostic tools in periodontitis and periimplantitis. As such, lateral flow quantitative active matrix metalloproteinase (aMMP)-8 POC testing is a potential such tool with sensitivity 75–85% and specificity 80–90%, in agreement with independent benchtop catalytic protease activity assays of aMMP-8. Hence, aMMP-8 can be considered as a key biomarker to be implemented in the new classification systems for periodontal and peri-implant diseases. Additional applications of this test include the detection prediabetes/diabetes, oral side-effects of head and neck cancer radiotherapy, and as a potential risk disease for COVID-19. This aMMP-8-technology could as well be implemented as a systemic medical biomarker, with applications in cardiovascular diseases, diabetes, obesity, bacteraemia, sepsis, meningitis, and pancreatitis.

**Anders Johansson** discussed the genetic diversity within the species *Aggregatibacter actinomycetemcomitans*, resulting in virulent, as well as harmless genotypes. A common feature of the virulent genotypes is an enhanced expression of a leukotoxin that profoundly affects leukocytes. A genetic marker for these genotypes is the *cagE* gene, present solely in a subgroup of serotype b that includes the JP2-genotype, as well as the majority of highly leukotoxic non-JP2 genotypes. The presence of these highly leukotoxic variants in subgingival biofilms is a strong risk marker for degenerative processes in the periodontium.

**Anna Dongari-Bagtzoglou** showed in a mouse model of infection that changes in oral mucosal microbial communities, in response to a sucrose-enriched diet, can lead to attenuation of *Candida* yeast virulence. These changes mainly involved overgrowth of *Lactobacillus johnsonii* which may curtail *Candida* virulence both by inhibiting its growth and by inhibiting the growth of potentially synergistic bacteria, such as enterococci. Her work presented in this meeting supported the concept that *Candida* pathogenesis should be viewed both in the context of a susceptible host and a virulence-conductive mucosal bacterial microbiota.

**Michel (Hyun) Koo** discussed the unique spatial structure of intact biofilms formed on human teeth, and their native state associated with severe childhood caries. The Koo group identified a 3D rotund-shaped architecture harboring multiple species precisely arranged in a corona-like structure with an inner core of *S. mutans* surrounded by outer layers of other bacteria. This spatial arrangement is mediated by active production of an extracellular polymeric scaffold. This architecture provides antimicrobial protection to the pathogen core and creates localized regions of acidic pH and severe enamel demineralization, suggesting this highly ordered community as the causative agent of caries. In other words, this precise biogeography in a polymicrobial community associated with human caries may modulate the pathogen positioning and virulence potential *in situ*.

**Jens Kreth** presented research on the oral commensal species *Corynebacterium durum* and *Streptococcus sanguinis*. Their specific relationship leads to a metabolic exchange of fatty acids from *C. durum* that influences *S. sanguinis* chain morphology and interaction with the host immune system. It provided a prime example of how individual members of the commensal oral microbiome interact on the cellular level to promote a healthy relationship with the host, defined as molecular commensalism.

**Grzegorz Bereta** indicated that several reports indicate that recombinant *P. gingivalis* peptidylarginine deaminase (PPAD) has endoarginine deimninase activity in contrast to the native mature enzyme purified from *P. gingivalis* showing strong preference for C-terminal Arg residues. He elaborated that this discrepancy may be related to the presence of N-terminal extension in recombinant PPAD which is absent from the native enzyme. To verify this, they directly compared native PPAD with recombinant variants of the enzyme with or without the N-terminal extension, and the latter had low activity for internal Arg and preferentially citrullinated the C-terminal Arg residue.

**Esra Sahingur** discussed about A20, an ubiquitin-editing enzyme which mainly functions as an endogenous regulator of inflammation through termination of NF-κB activation as part of negative feedback loop. A20 is known to interact with substrates that reside downstream of immune sensors including various receptors. Due to its pleiotropic functions as an ubiquitin binding protein, de-ubiquitinase and ubiquitin ligase and its versatile role in various signaling pathways, aberrant A20 levels are associated with numerous conditions, such as rheumatoid arthritis, diabetes, systemic lupus erythematosus, and inflammatory bowel disease. Similarly, the Sahingur group has recently established A20 as an essential regulator of inflammation in the oral cavity. The presentation provided an overview of how the dysregulation of NF-κB/A20 axis can affect cellular responses and provide perspectives how this pathway can be exploited to alleviate periodontal inflammation and maintain oral mucosa tissue homeostasis.

**Aleksander Grabiec** spoke on the synergistic activation of gingival fibroblasts by the inflammatory tissue environment and oral pathogens, as a potential pathomechanism for periodontitis. While the manipulation of immune and gingival epithelial cells by oral pathogens has been widely investigated, gingival fibroblast responses have been understudied in the context of the local microenvironment of the inflamed gingival tissue. Their data reveals that oral pathogens, such as *P. gingivalis, Fusobacterium nucleatum* and *Filifactor alocis*, synergize with tumor necrosis factor (TNF) in a toll-like receptor (TLR2)-dependent manner, to amplify fibroblast inflammatory responses, thereby promoting gingival tissue damage.

**Katarzyna Lagosz-Cwik** discussed the effects of DNA methyltransferase (DNMT) inhibitors on gingival fibroblasts. Epigenetic regulatory mechanisms are dysregulated in the gingival tissue during the development of periodontitis, and compounds that target chromatin-modifying enzymes, such as histone deacetylase inhibitors, may display clinical potential as host modulation agents. DNMT inhibitors protect against bone resorption and disruption of the epithelial barrier in periodontitis models, but their therapeutic potential may be limited due to their effects on gingival fibroblast viability and exaggeration of inflammatory responses. Nonetheless, DNMT inhibitors may be a useful tool for studying the role of DNA methylation in the regulation of expression of inflammatory mediators, such as the Th17 chemokine CCL20 and MMPs.

**Aleksandra Wielento** discussed how PPAD activity influences TLR2-dependent host cell responses to *P. gingivalis* infection. The results unambiguously revealed that PPAD activity is required for TLR2 activation, induced by citrullinated fimbriae (possibly type I). Citrullinated ligands activate NF-kB and MAP-kinase-dependent signaling pathways in primary human gingival fibroblasts.

**Juliana Barreto de Albuquerque** highlighted that although the oral cavity is the first contact of ingested microorganisms with the host, most studies focus on the immune response in the lower gastrointestinal tract and associated lymphoid organs. Their work demonstrated the relevance of the oral mucosa as a gateway for pathogen dissemination and activation of the adaptive immune system in the oral mucosa-draining lymph nodes. Furthermore, ingested microorganisms lead to the activation of CD8^+^ T cells in oral mucosa-draining lymph nodes, which can then migrate to other lymphoid and non-lymphoid organs, especially spleen, oral mucosa and lung, and thereby contribute to systemic protection.

**Jeff Ebersole** highlighted that a feature of periodontitis is the detection of adaptive immune responses to members of the oral microbiome that show specificity and changes with disease and treatment. Variations in these antibody responses are noted across the population and affected by aging, but it is still unclear as to how these relate to disease risk and expression. A non-human primate model of experimental periodontitis was used to track local microbiome changes associated with a repertoire of immunoglobulin genes in gingival tissues. Interfacing an array of microbes and host responses clearly discriminated between the transition from health to disease and resolution of lesions. These results support a major importance of adaptive immune responses in the kinetics of periodontal lesion formation and highlight the effects of aging on the repertoire of Ig genes that may relate to the increased prevalence and severity of periodontitis with age.

**George Hajishengallis** talked about his collaborative work on complement and periodontitis, which started more than 10 years ago in preclinical models and recently reached clinical trials in human patients. Specifically, in collaboration with his colleague at Penn, John Lambris, they first showed in mice that complement is involved both in the development of microbial dysbiosis and the destructive inflammation that drives alveolar bone loss. They subsequently showed that pharmacological blockade of the central complement component C3 in non-human primates protects them from induced or naturally occurring periodontitis. The C3 inhibitor used was a peptidic drug AMY-101 invented by John Lambris. More recently, in a placebo-controlled, double-blind phase 2a clinical trial in adult patients, led by Hatice Hasturk at Forsyth Institute, local administration of AMY-101 resulted in significant and sustainable reduction in periodontal inflammation without adverse events.

**Hatice Hasturk** discussed in detail the findings of this recent phase 2a clinical trial. Specifically, AMY-101 significantly reduced clinical indices that measure gingival inflammation (gingival index and bleeding on probing). Moreover, AMY-101 significantly reduced the gingival crevicular fluid levels of matrix metalloproteinases−8 and−9, which are indicators of inflammatory tissue destruction. The drug was administered once-per-week *via* intragingival injections and its therapeutic effects persisted for at least 3 months post-treatment. These clinical findings suggest that C3-targeted therapy has high potential as a novel host modulatory therapy capable of resolving inflammation and providing opportunity for the tissues to return to homeostasis. The collaborative team is currently planning a phase 3 clinical trial.

**Silvia Uriarte** started with an overview of several neutrophil functions that have been previously tested in the context of *F. alocis* challenge. She then described her more recent work that focused on the effect of this organism on neutrophil lifespan. Their data showed that interactions between human neutrophils and *F. alocis* result in upregulation of anti-apoptotic proteins, dampening of caspase-3 activation, and delayed DNA fragmentation. Furthermore, *F. alocis*-challenged neutrophils were more efficient at phagocytosis and mounting a robust respiratory burst response when exposed to opsonized *Staphylococcus aureus* than control-treated cells. In summary, this prolonged lifespan and ability to retain functional capacity could result in increased neutrophil-driven inflammation, which is a hallmark of periodontitis.

**Georgios Belibasakis** also discussed about *F. alocis* and presented how phylogenetic analysis of 10 genome-sequenced strains led to the discovery of a new member of the repeats-in-toxins (RTX) family. The ftxA gene was expressed by 60% of the strains.

**Nagihan Bostanci** detailed how proteomics led to the discovery of thousands of proteins in gingival tissues and saliva and facilitated our understanding of inter-individual variability in periodontal inflammation and disease. Proteome mapping by application of contemporary proteomics was highlighted as a tool to study biological susceptibility to gingivitis, anticipating that it may help diagnose early and prevent destructive periodontal disease.

**Filippos Kardaras** introduced “SCHINOS,” an ongoing study with a goal to examine for the first time the holistic effects of Chios mastic on the oral microbiome and to elucidate mechanistically the effects of this natural product on the host immune response after oral administration. Next Generation Sequencing techniques will be combined with state-of-the-art bioinformatics analyses and applied in patients with gingivitis before and after administering Chios mastic and mastic oil.

## Conclusion

**Figure 1:**
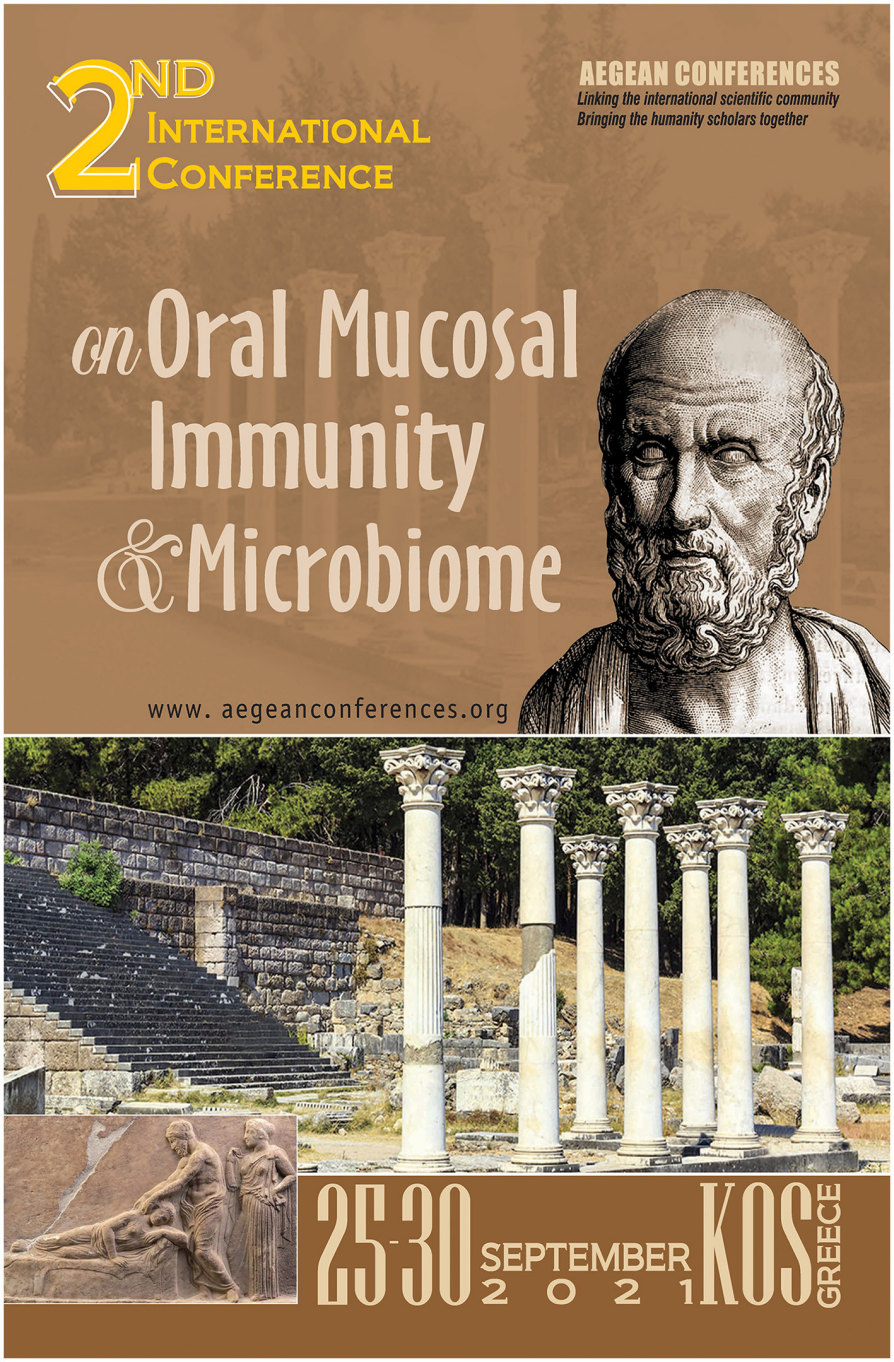
The poster announcement of the 2nd International Conference on Oral Mucosal Immunity and Microbiome.

**Figure 2:**
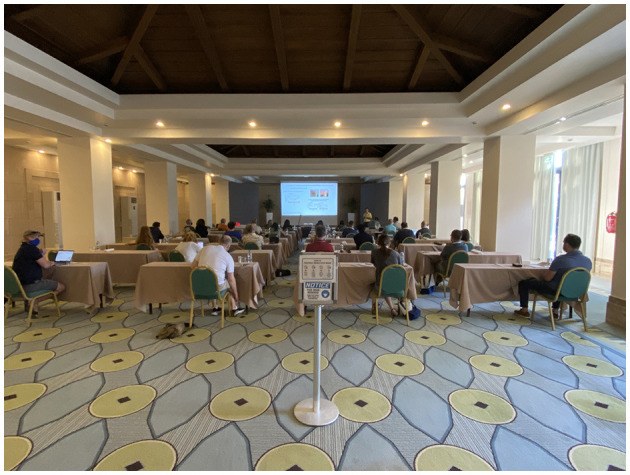
Lively talks and discussions during the Conference, under strict hygiene regulations due to the COVID-19 pandemic.

This paper summarizes the research presented at the 2nd OMIM (Figure 1). In the proceedings of the inaugurating OMIM conference [1], we emphasized that cross-disciplinary research is the way forward for understanding the microbial and immune components of oral disease and for achieving predictable preventive, diagnostic, and treatment strategies. Three years later, and despite the intervening pandemic (Figure 2), tremendous progress from inter-disciplinary collaborations has been made not only at the fundamental level of mechanistic understanding of pathogenic mechanisms but also at the translational level, as evidenced by improved diagnostics and emerging host-modulation treatments for human patients. The latter provides us with hope that the gaps between clinically relevant research findings and actual clinical practice will be overcome in the near future.

## Author Contributions

All authors listed have made a substantial, direct, and intellectual contribution to the work and approved it for publication.

## Funding

The 2nd OMIM Conference was sponsored by the Aegean Conferences organization, the Frontiers organization, Colgate, Nanotemper Technologies and Bentley Polska.

## Conflict of Interest

The authors declare that the research was conducted in the absence of any commercial or financial relationships that could be construed as a potential conflict of interest.

## Publisher's Note

All claims expressed in this article are solely those of the authors and do not necessarily represent those of their affiliated organizations, or those of the publisher, the editors and the reviewers. Any product that may be evaluated in this article, or claim that may be made by its manufacturer, is not guaranteed or endorsed by the publisher.
